# Supplementary figures and images for: PDGFRA defines the mesenchymal stem cell Kaposi’s sarcoma progenitors by enabling KSHV oncogenesis in an angiogenic environment
Source: PLoS Pathog. 2019 Dec 27;15(12):e1008221. doi: 10.1371/journal.ppat.1008221 (PMC6980685; doi:10.1371/journal.ppat.1008221)

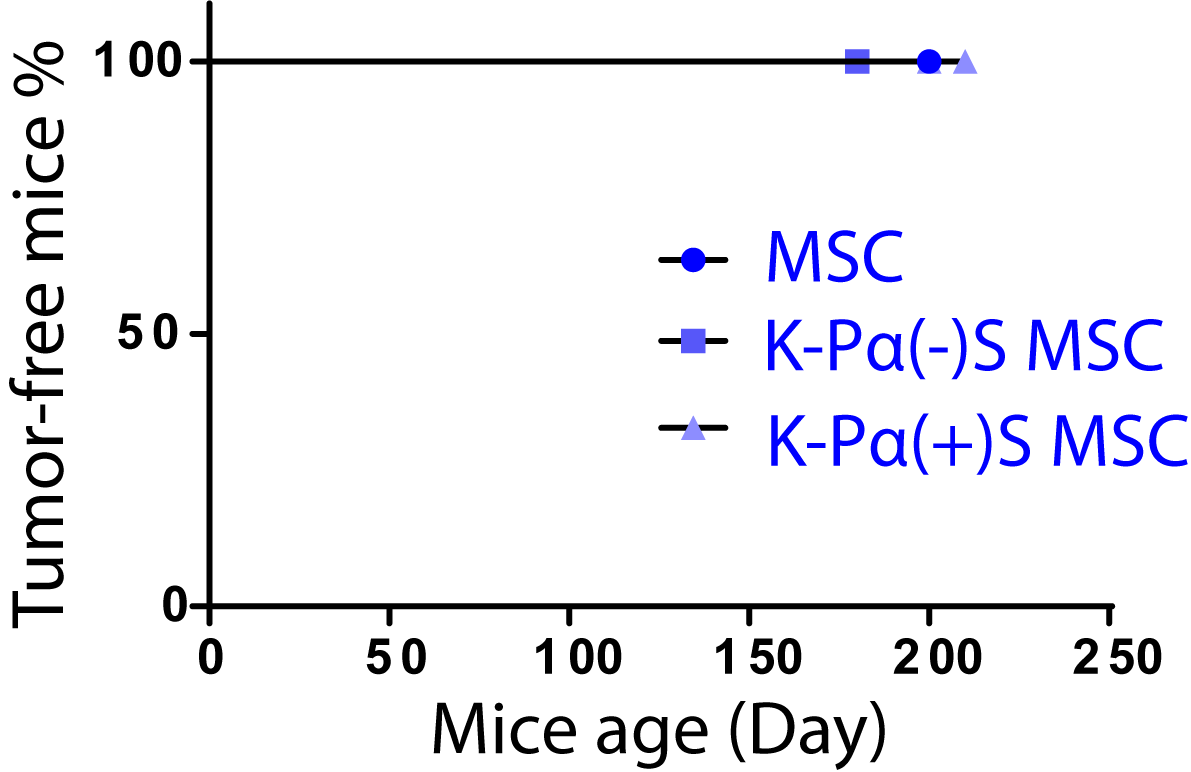

Supplement: S1 Fig — (TIF) [file ppat.1008221.s001.tif]

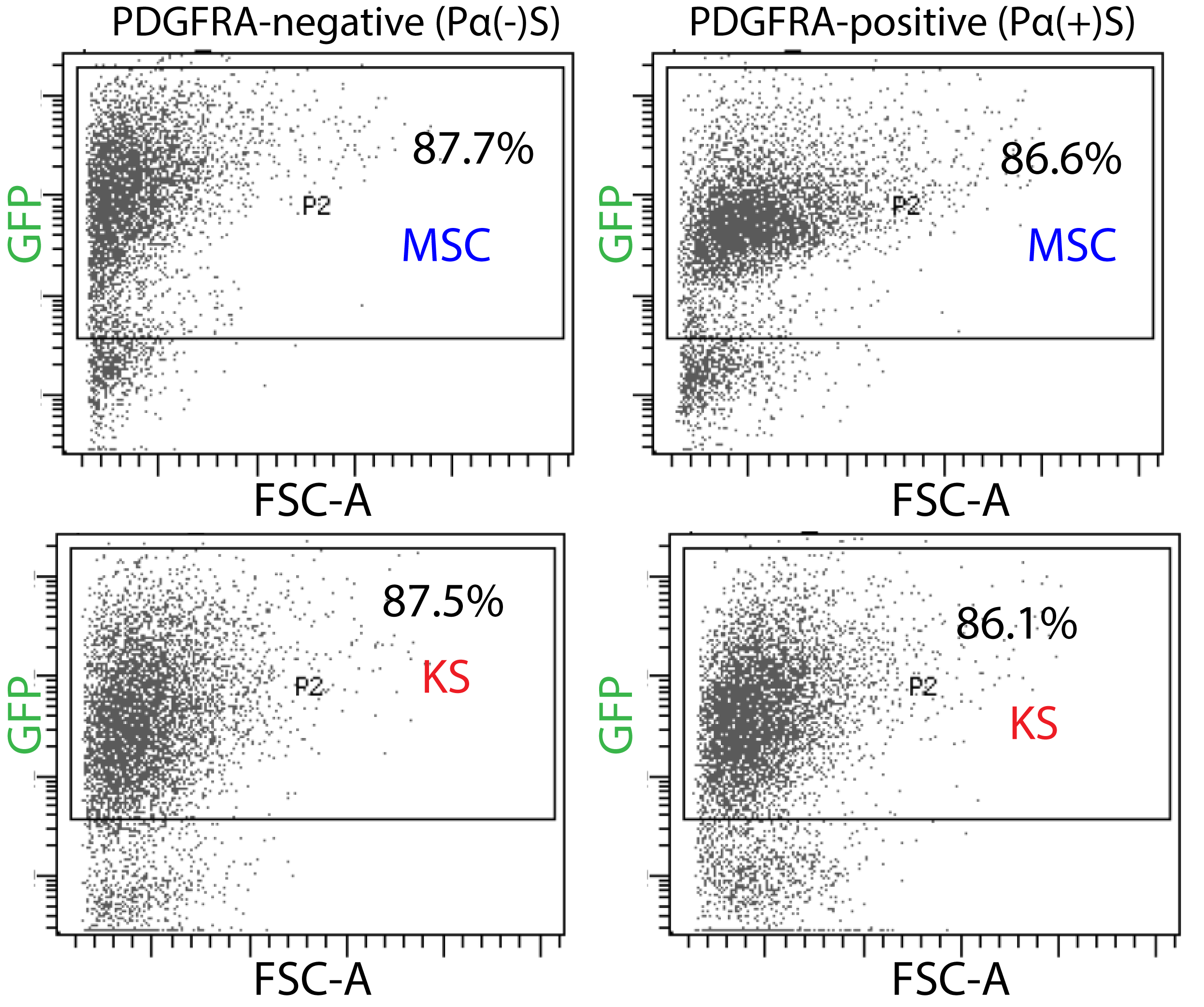

Supplement: S2 Fig — (TIF) [file ppat.1008221.s002.tif]

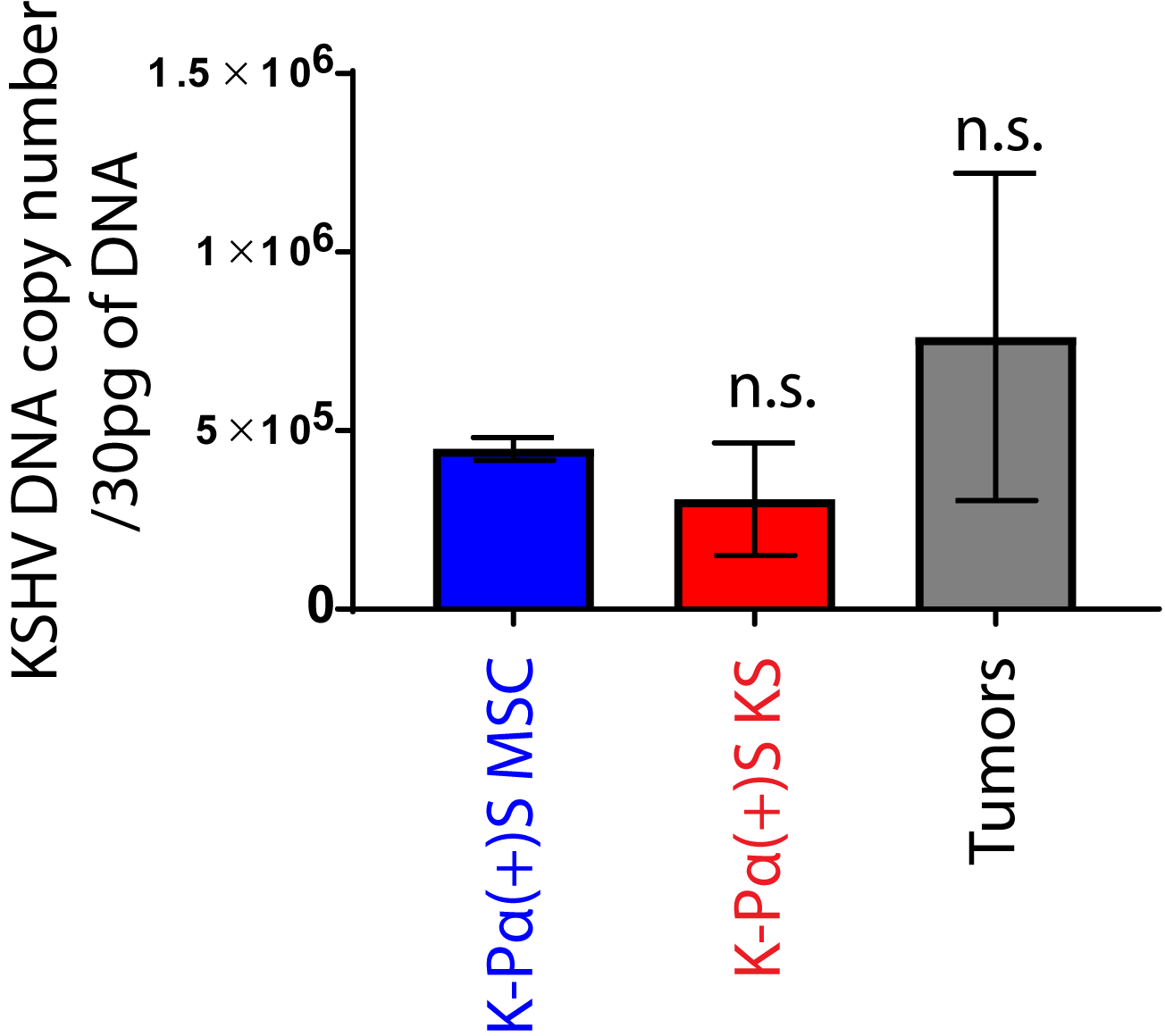

Supplement: S3 Fig — (TIF) [file ppat.1008221.s003.tif]
